# Untreated Active Tuberculosis in Pregnancy with Intraocular Dissemination: A Case Report and Review of the Literature

**DOI:** 10.1155/2015/370462

**Published:** 2015-11-26

**Authors:** Shadi Rezai, Stephen LoBue, Daniel Adams, Yewande Oladipo, Ramses Posso, Tiffany Mapp, Crystal Santiago, Manisha Jain, William D. Marino, Cassandra E. Henderson

**Affiliations:** ^1^Department of Obstetrics and Gynecology, Lincoln Medical and Mental Health Center, Bronx, NY 10451, USA; ^2^St. George's University, School of Medicine, Grenada; ^3^Coney Island Hospital, Pulmonary Medicine, Brooklyn, NY 11235, USA; ^4^Cornell Medical College, NY 10065, USA

## Abstract

*Background.* Tuberculosis (TB) is a disease that affects hundreds of millions of people across the world. However, the incidence in developed countries has decreased over the past decades causing physicians to become unfamiliar with its unspecific symptoms. Pregnant individuals are especially difficult because many symptoms of active TB can mimic normal physiological changes of pregnancy. We present a case report of a 26-year-old multiparous woman, G4P3003, at 38-week gestation with a history of positive PPD who emigrated from Ghana 6 years ago. She came to the hospital with an initial complaint of suprapubic pain, pressure, and possible leakage of amniotic fluid for the past week. Patient also complained of a productive cough for the past 3 to 4 months with a decrease in vision occurring with the start of pregnancy. Visual acuity was worse than 20/200 in both eyes. Definitive diagnosis of active TB was delayed due to patient refusal of chest X-ray. Fortunately, delay in diagnosis was minimized since patient delivered within 24 hours of admission. Active TB was confirmed with intraocular dissemination. Patient had optic atrophy OS (left eye) and papillitis, choroiditis, and uveitis OD (right eye) due to TB infiltration. Fetus was asymptomatic and anti-TB therapy was started for both patients.

## 1. Introduction 

According to the most recent World Health Organization (WHO) data from 2014, around 3.3 million women contract tuberculosis (TB) a year with a total mortality of about 510,000 [1]. Out of 510,000 people who died, 180,000 were HIV positive [1]. Worldwide, TB is the third leading cause of morbidity and premature mortality in women of reproductive age from 15 to 44 years old [2–4].

However, approximately one-third of the world's population or approximately 900 million women have latent tuberculosis infection (LTBI) [1]. Pregnant women with LTBI are more likely to progress to active tuberculous disease than men [5].

The prevalence of active TB among pregnant women ranges from 0.06% to 0.25% in low-burden countries compared to 0.07% and 0.5% in high-burden countries. Prevalence was found to increase in high-burden countries to 0.7% and 11% when coinfected with HIV [6].

In 2014, Southeast Asia and the Western Pacific Regions had the greatest incidence of TB, accounting for 58% of new cases globally [1]. However, Africa carried the most severe burden, with an average of 281 cases per 100,000 compared with a global average of 133 per 100,000 [1]. Particularly, the prevalence of TB in Ghana is very high with 282 infected with TB per 100,000 [1].

Although the prevalence of TB has decreased in the United States, immigrant populations from high-burden areas as well as those with immunodeficiency have significantly increased risks [1, 7].

## 2. Presentation of Case

A 26-year-old multiparous woman from Ghana, with a history of positive PPD (purified protein derivative) test and gestational diabetes, presented to labor and delivery at 38-week gestation with complaints of suprapubic pain, pressure, and possible leakage of amniotic fluid.

The patient's suprapubic pain had occurred for a week and was associated with vomiting. The patient also complained of a productive cough for the past 3 to 4 months with a decrease in vision occurring at the start of pregnancy. She reported good fetal movement and denied vaginal bleeding. She also denied the use of alcohol, tobacco, and illicit drugs. The patient had a history of positive PPD with a normal chest X-ray in 2009. Treatment never occurred as the patient was lost to follow-up.

Physical examination revealed a frequent productive cough with urinary incontinence. The patient had a visual acuity worse than 20/200 in each eye and was referred to ophthalmology. Ophthalmology diagnosed her with optic atrophy OS (left eye) and papillitis, choroiditis, and uveitis OD (right eye). She was prescribed ophthalmic prednisolone and cyclopentolate for the uveitis and timolol for secondary glaucoma due to the uveitis. However, the patient refused all eye medications stating that headaches and eye pain occurred with use.

The patient was also evaluated by pulmonology for chronic cough. Initial imaging was delayed due to patient refusal. The mother believed that imaging would be harmful to the fetus and would not consent to the procedure.

**Figure 1:**
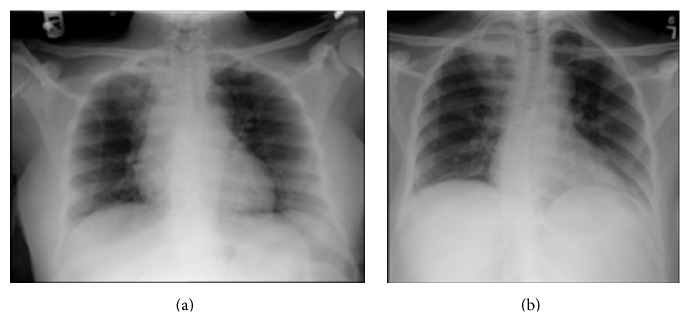
Chest imaging prior to anti-TB therapy and during TB therapy. (a) CXR showing nodular opacity in the apex of the lung fields and (b) CXR showing increased density in the right upper lobe.

Day one after admission the patient delivered a healthy infant of 2960 grams at 38 weeks via vacuum assisted vaginal delivery. APGAR score was 9/9 with no perinatal complications. After the patient delivered, a chest X-ray (CXR) showed right nodular opacity in the apex of the lung field measuring 1.6 × 1.0 cm (Figure 1(a)).* Mycobacterium tuberculosis* was confirmed by positive AFB smear and culture leading to the diagnosis of active TB.

The baby was isolated from the mother and fed with formula due to increased likelihood of TB transmission. Physical examination performed on the newborn revealed no anatomic, visual, or neurologic deficits. As a safety precaution, the infant was initiated on isoniazid oral suspension of 0.1 mg and pyridoxine oral suspension of 29 mg daily (10 mg/kg/day).

Immediately following maternal diagnosis of active TB, the patient was isolated and started on an antituberculosis regimen comprising of isoniazid and pyridoxine 300 mg once daily, rifampin 300 mg twice daily, pyrazinamide 500 mg three times daily, and ethambutol 1,200 mg daily to be completed on a 6-month course. However, ethambutol was discontinued due to the patient's history of left optic nerve atrophy, which may progress due to ethambutol toxicity.

Prior to being discharged, a CXR was done on April 7, 2015, which revealed increased density in the right upper lobe that appeared to be more prominent compared to the previous imaging (Figure 1(b)). On April 13, 2015, the patient was cleared for discharge after the documentation of three consecutive partially negative AFB smears and culture. She was released to the care of a public health advisor at the Department of Health who will provide follow-up care.

## 3. Discussion

Tuberculosis (TB) is a contagious, airborne pathogen, listed as the second leading cause of death from an infectious agent [1]. Its mortality rate as an infectious agent is second only to HIV [1]. TB is also one of the top causes of mortality in women from 15 to 44 years old [2–4]. However, within this demographic, the most susceptible group of women are pregnant individuals. Pregnant women with latent tuberculosis infection (LTBI) are more likely to progress to active tuberculous disease than men [5].

The increased rate of progression of LTBI to active infection is supported by immunological changes associated with pregnancy. For one, pregnancy is linked with upregulation of potent anti-inflammatory hormones such as cortisol [8]. A progressive increase of circulating CRH, ACTH, and free cortisol levels has been documented in the third trimester [8]. One study documented a gradual increase in total plasma cortisol and 24-hour urinary free cortisol with levels peaking during the third trimester to levels threefold higher than those in nonpregnant controls [9].

Increased levels of glucocorticoids suppress innate and cellular immune responses [10–13]. Previous studies have indicated that glucocorticoids inhibit T-helper 1 (Th1) and enhance T-helper 2 (Th2) cytokine secretion, thus impeding the effectiveness of cellular immunity on intracellular organisms such as* Mycobacterium tuberculosis* [10–13]. A downregulation of Th1 inhibits the production of interferon-gamma (IFN-y) and Interleukin 12 (IL-12). IFN-y is a vital macrophage activating cytokine involved in cellular immunity against* Mycobacterium tuberculosis* [14]. Individuals become highly susceptible to mycobacteria infection when IFN-y production is absent or decreased [15]. A decrease in IL-12 also hinders activation of natural killer cells which are also important for fighting intracellular pathogens [4, 16].

Although progression of latent tuberculosis infection (LTBI) to an active infection is more common in pregnancy, the clinical diagnosis is often delayed. For one, manifestations of active TB may go unnoticed due to overlap of normal physiological changes in pregnancy [17, 18]. Symptoms such as fatigue, sweating, shortness of breath, and low grade fever are similar to the physiological symptoms seen in pregnancy [17, 18].

However, patients from TB endemic nations should have increased clinical suspicion for active or latent TB. Endemic areas include India, Indonesia, Nigeria, Ghana, South Africa, Pakistan, and China [1]. Our patient had many nonspecific symptoms common in pregnancy. However, the chronic productive cough coupled with endemic travel history led us to have a high clinical suspicion of TB.

Nevertheless, a definitive diagnosis was delayed. Chest radiographs with abdominal shields are often delayed until after delivery due to maternal concerns for fetal health [19]. Our patient also adamantly refused imaging for concern of fetal safety. However, a tuberculin skin test is a safe and effective diagnostic tool for TB in pregnancy. Patient exhibiting night sweats, evening pyrexia, hemoptysis, weight loss, history of travel from an endemic area, and chronic productive cough for over 3 weeks duration should have a tuberculin skin test [20, 21]. Most immigrants from TB endemic countries in Asia and Africa were vaccinated against certain strains of TB with the Bacillus Calmette-Guerin (BCG) vaccine. BCG vaccine will produce a false positive PPD, a negative QuantiFERON, and a negative chest X-ray [21, 22]. Unfortunately many physicians are unaware that the protocol for screening and treatment of BCG vaccinated patients is identical to the protocol for non-BCG vaccinated patients [22].

A delay in treatment may allow active pulmonary TB to disseminate, becoming extrapulmonary tuberculosis (EPTB). EPTB may affect cardiovascular system, skin, central nervous system, gastrointestinal tract, genitourinary tract, and eyes [23]. The prevalence of EPTB is not well documented but approximately 15% of the cases are extrapulmonary tuberculosis in low incidence countries such as the United States and Great Britain [24]. Once again EPTB symptoms may be vague including fatigue, malaise, nausea/vomiting, and anorexia. Many of these symptoms overlap with pregnancy and are difficult to discern from an activated TB infection [4, 25].

The incidence of ocular TB is unknown due to lack of uniform diagnostic criteria [26]. Initially ocular TB was considered to be very rare but a study in Spain from 1997 showed that 18 out of 100 people with confirmed systemic tuberculosis had ocular manifestations [27]. Ocular lesions included choroiditis, papillitis, retinitis, vasculitis, dacryoadenitis, and scleritis [27]. However, more recent studies in other countries found intraocular TB to be much lower. A study in Japan found a rate of uveitis due to TB to be 6.9% out of 189 patients [28]. Yet in China only 4% of uveitis was due to TB [29]. A study in Riyadh, Saudi Arabia, had an incidence of 10.5% of cases [30]. However, larger studies in India found the incidence of ocular TB to be significantly lower with variability occurring among the same center. One study in South India of 1,005 patients with active pulmonary and extrapulmonary TB reported ocular manifestation in only 1.39% of patients [31]. Another study from the same center reported ocular tuberculosis contributed to only 0.39% of uveitis seen in 1,273 patients [32].

The most common clinical presentation of ocular TB is posterior uveitis [27]. Other common ocular symptoms which have been noted include anterior uveitis, intermediate uveitis, retinitis, choroiditis, retinal vasculitis, optic neuropathy, neuroretinitis, endophthalmitis, and panophthalmitis [27]. The involvement of the optic nerve from TB can manifest as an optic nerve tubercle, papillitis, papilledema, optic neuritis, retrobulbar neuritis, neuroretinitis, or optochiasmatic arachnoiditis [26]. In this case report, our patient had papillitis, choroiditis, posterior uveitis OD (right eye), and optic atrophy OS (left eye). The funduscopic examination results were consistent with pathology caused by hematogenous dissemination of TB to the eyes, with subsequent posterior segment inflammation and optic neuropathy. MRI of the head without IV contrast revealed no dissemination to the brain.

Yet, one of the most concerning complications with EPTB during pregnancy is vertical transmission to the fetus. Active TB in pregnancy is associated with increased fetal risks including prematurity, low birth weight, growth retardation, and low Apgar scores [4, 5, 33].

One study in Sub-Saharan Africa analyzed 107 pregnant women with TB, of which 50% had systemic TB, showing that 46% of newborns were premature, 66% had low birth weight, and 49% had intrauterine growth restriction [34]. 16% also had vertical transmission of TB from mother to infant [34]. However, it is important to note that, of the 107 patients, 82 were coinfected with HIV-1. Although HIV-1-infected mothers and their exposed newborns had significantly lower CD4 counts, there was no association between perinatal maternal viral load, CD4 count, and vertical transmission of TB [34].

However, another study in India noted a vertical transmission of TB in only 9% of infants born to mothers of active TB and HIV coinfection [35]. This study found TB to be strongly associated with postpartum maternal and infant death [35]. Smaller gestational sizes and increased infant morbidity and mortality were also seen from studies in South Asia and Mexico [33, 36–38]. One case report documented a disseminated TB inducing a spontaneous abortion [39].

Nevertheless, the fetus may be asymptomatic even in disseminated TB [40, 41]. Our patient delivered a healthy, full term infant, with no complications. Regardless of the infant being asymptomatic, treatment should begin immediately for both the mother and infant.

Almost half of children born to mothers with active TB will become infected within the first year if they are not given appropriate chemoprophylaxis [42]. Asymptomatic infants should be started on isoniazid (INH) 10 to 15 mg/kg po once/daily and discharged home at the normal time. Infants who will be breastfed should receive pyridoxine 1 to 2 mg/kg once daily [42]. A PPD skin test should be done when the baby is 3 or 4 months of age. If the baby screens negative and the mother has been compliant with her course of anti-TB treatments, INH treatment for the baby is discontinued [42]. However, if the baby screens positive, chest X-ray and cultures for acid-fast bacilli are taken. If active disease is excluded, treatment with INH is continued for a total of 9 months. If cultures become positive for TB at any time, the baby should be treated for active TB.

Treatment of TB in neonates slightly varies depending on whether TB is congenital or acquired after birth. Treatment of congenital TB requires isoniazid 10 to 15 mg/kg po, rifampin 10 to 20 mg/kg po, pyrazinamide 30 to 40 mg/kg po, and an aminoglycoside such as amikacin [42]. Pyridoxine is also given in neonates exclusively breastfed [42]. Acquired TB after birth requires treatment once/day with isoniazid 10 to 15 mg/kg po, rifampin 10 to 20 mg/kg po, and pyrazinamide 30 to 40 mg/kg [42]. A fourth drug such as ethionamide, ethambutol, or an aminoglycoside can be added if drug resistance or TB meningitis is suspected [42]. After the first 2 months of treatment, all drugs are stopped besides isoniazid and rifampin which are continued for a 6- to 12-month course depending on the disease category [42].

Most first-line medications used in the treatment of TB are proven to be safe for use during the antenatal and postpartum breastfeeding period [17]. Treatment of EPTB follows the same scheme as active pulmonary TB. Isoniazid (INH), rifampin (RIF), and ethambutol (EMB) are used daily for 2 months. After 2 months, ethambutol is discontinued. INH and RIF are administered daily for 7 months for a total of 9 months of treatment. Streptomycin is potentially ototoxic to the fetus and should not be used unless rifampin is contraindicated [42]. Also, pyrazinamide (PZA) is not recommended to be used because its effect on the fetus is unknown.

However, due to optic neuropathy associated with ethambutol [26], our patient was started on ethambutol and discontinued to prevent any further damage to the eyes. Some slow responding cases of TB require a prolonged course of 12 months of pharmacotherapy [17]. In addition, women with TB involvement of the meninges, pericardium, or eye may benefit from the addition of oral corticosteroids or ophthalmic corticosteroids to the treatment regimen [26].

## 4. Conclusions

TB is also one of the top causes of mortality of women of reproductive ages from 15 to 44 [2–4]. However, within this demographic, the most susceptible group of women are pregnant individuals. Pregnant women with latent tuberculosis infection (LTBI) are more likely to progress to developing active tuberculous disease than men [5]. Although progression of latent tuberculosis infection (LTBI) to an active infection is more common in pregnancy, the clinical diagnosis is often delayed. For one, manifestations of active TB may go unnoticed due to overlap of normal physiological changes in pregnancy [17, 18]. Symptoms such as fatigue, sweating, shortness of breath, and low grade fever are similar to the physiological symptoms seen in pregnancy [17, 18].

Our patient was an immigrant from Ghana who presented with vague symptoms. She had suprapubic pain associated with nausea, vomiting, chronic productive cough for 3 to 4 months, and a significant decrease in vision bilaterally. Radiological imaging was initially delayed 24 hours until after gestation due to maternal fear of infant safety. One day into the postpartum a diagnosis of active TB with intraocular dissemination was supported based on findings of chest X-ray, positive QuantiFERON test, and positive AFB culture and smear.

Based on this case report we present several learning objectives. For one, clinicians should be alert for tuberculosis in women that have lived in endemic areas including India, Indonesia, Nigeria, Ghana, South Africa, Pakistan, and China. A high clinical suspicion for TB should be employed for all pregnant females emigrating from these endemic areas. Secondly the importance of radiologic imaging needs to be explained to the patient in order to prevent diagnostic delay. Delay is most often due to cultural and communication barriers. Thus, more time needs to be spent reassuring and addressing the concerns of the mother that may result in any delay in treatment. Evidence has shown that prenatal diagnosis and treatment of TB result in a better outcome for the mother and infant [42]. Lastly, all first-line medications used in the treatment of TB are proven to be safe for use during the antenatal and postpartum breastfeeding period [5]. Asymptomatic infants should always be treated if TB is in question with the mother.
